# Increased Plant Carbon Translocation Linked to Overyielding in Grassland Species Mixtures

**DOI:** 10.1371/journal.pone.0045926

**Published:** 2012-09-25

**Authors:** Gerlinde B. De Deyn, Helen Quirk, Simon Oakley, Nick J. Ostle, Richard D. Bardgett

**Affiliations:** 1 Soil and Ecosystem Ecology Laboratory, Lancaster Environment Centre, Lancaster University, Lancaster, United Kingdom; 2 Department of Soil Quality, Wageningen University, Wageningen, The Netherlands; 3 Plant-Soil Interactions Group, Centre for Ecology and Hydrology, Lancaster Environment Centre, Lancaster, United Kingdom; University of Zurich, Switzerland

## Abstract

Plant species richness and productivity often show a positive relationship, but the underlying mechanisms are not fully understood, especially at the plant species level. We examined how growing plants in species mixture influences intraspecific rates of short-term carbon (C-) translocation, and determined whether such short-term responses are reflected in biomass yields. We grew monocultures and mixtures of six common C3 grassland plant species in outdoor mesocosms, applied a ^13^C-CO_2_ pulse *in situ* to trace assimilated C through plants, into the soil, and back to the atmosphere, and quantified species-specific biomass. Pulse derived ^13^C enrichment was highest in the legumes *Lotus corniculatus* and *Trifolium repens*, and relocation (i.e. transport from the leaves to other plant parts) of the recently assimilated ^13^C was most rapid in *T. repens* grown in 6-species mixtures. The grass *Anthoxanthum odoratum* also showed high levels of ^13^C enrichment in 6-species mixtures, while ^13^C enrichment was low in *Lolium perenne*, *Plantago lanceolata* and *Achillea millefolium*. Rates of C loss through respiration were highest in monocultures of *T. repens* and relatively low in species mixtures, while the proportion of ^13^C in the respired CO_2_ was similar in monocultures and mixtures. The grass *A. odoratum* and legume *T. repens* were most promoted in 6-species mixtures, and together with *L. corniculatus*, caused the net biomass increase in 6-species mixtures. These plant species also had highest rates of ^13^C-label translocation, and for *A. odoratum* and *T. repens* this effect was greatest in plant individuals grown in species mixtures. Our study reveals that short-term plant C translocation can be accelerated in plant individuals of legume and C3 grass species when grown in mixtures, and that this is strongly positively related to overyielding. These results demonstrate a mechanistic coupling between changes in intraspecific plant carbon physiology and increased community level productivity in grassland systems.

## Introduction

Primary production often increases with plant species richness, as demonstrated in a number of biodiversity experiments [1], [2]. Despite numerous studies on this topic, we still lack an understanding of the mechanisms that explain this positive plant biomass response to increased plant diversity. Several mechanisms have been proposed, involving both abiotic and biotic factors. For example, plant species grown in mixture can complement each other in their uptake of soil nutrients in time and/or space [3], [4], [5], and the chemical forms of soil nutrients that they access [6], [7], [8], and also dilution of plant species specific pathogens in species mixtures can contribute to positive diversity-productivity relationships [9]. It is generally recognised that legumes provide an additional nitrogen (N) input to soil by N_2_-fixation from the atmosphere. This provides legumes with a complementary N source as compared to non-legumes, and the subsequent decomposition of the N-rich roots enables higher productivity of non-legume species present within the plant community [10], [11], [12]. This in turn may benefit legume species through community level complementarity feedbacks to nutrient use efficiency, but the mechanisms involved remain poorly resolved.

It has been proposed that, when in mixtures, plants use soil nutrients more efficiently, thereby producing more biomass per unit of nutrient in their tissues, as shown for N by Fargione et al. [12] and by van Ruijven and Berendse [13]. Increased biomass production in mixtures has also been attributed to larger size or density of the component plant species [14]. For instance, Marquard et al. [14] found that plant species richness correlated positively with plant biomass due to increased density of the plants in plant species mixtures, and that variation in plant community biomass at different levels of species richness was related to the size of individual plants. Recently, it was also shown that grass species show plasticity in their morphological traits and nutrient and light acquisition in response to plant species richness and presence of legumes [15]. In that study grass species were found to become taller, with longer leaves and larger specific leaf area, and accessed other sources of N with increasing species richness and presence of legumes. Legume species have also been found to be plastic in their morphological and physiological traits including N-fixation in response to plant community diversity, albeit in a species-specific way [16]. Apart from altered morphology and N acquisition, the rate of carbon (C) assimilation and transfer (referred to as translocation or relocation in this paper) can be plastic in plant species, as demonstrated in grass species in response to the presence or absence of shrubs [17], or for legumes in response to functional groups of soil biota [18]. Moreover, intraspecific diversity can be important for the strength of interactions between species, which illustrates that intraspecific variances deserve attention in the context of species diversity-ecosystem functioning relations [19]. However, whether and how individuals of different plant species change their C translocation traits in response to growing together in species mixtures, and how such intraspecific responses affect their contribution to overall plant production in species mixtures, has not yet been tested.

The overall aim of this study was to examine how growing in a mixture influences intraspecific rates of short-term C translocation in six temperate grassland plant species, and determine whether such short-term responses are reflected in overall biomass yields. Specifically, we hypothesised that plant species with the highest short-term C-translocation rates also yield most biomass, and that this effect is enhanced when plants are grown in mixtures. This would mean that individuals in plant species mixtures are larger C sinks than in monoculture, and this can only be true if the newly assimilated C is translocated from the leaves to other plant parts that support plant biomass production over longer timescales (days to weeks before the C investment to belowground plant parts is notable in aboveground yields), rather than being respired back to the atmosphere. To test our hypothesis we designed a field-based grassland mesocosm experiment using two grass, two forb and two legume species, and grew them all in monocultures and in 6-species mixtures starting from seedlings, so that individuals of each species could be investigated in both community settings at the same planting density. We used an *in situ* ^13^C-CO_2_ pulse chase approach [20] to trace recent assimilated C in plants and soil respiration, in order to compare intraspecific rates of C translocation, and measured plant biomass yields at the end of the experiment, to investigate species specific biomass, in species mixtures and monocultures.

## Results

### Vegetation Relative Yield

**Figure 1:**
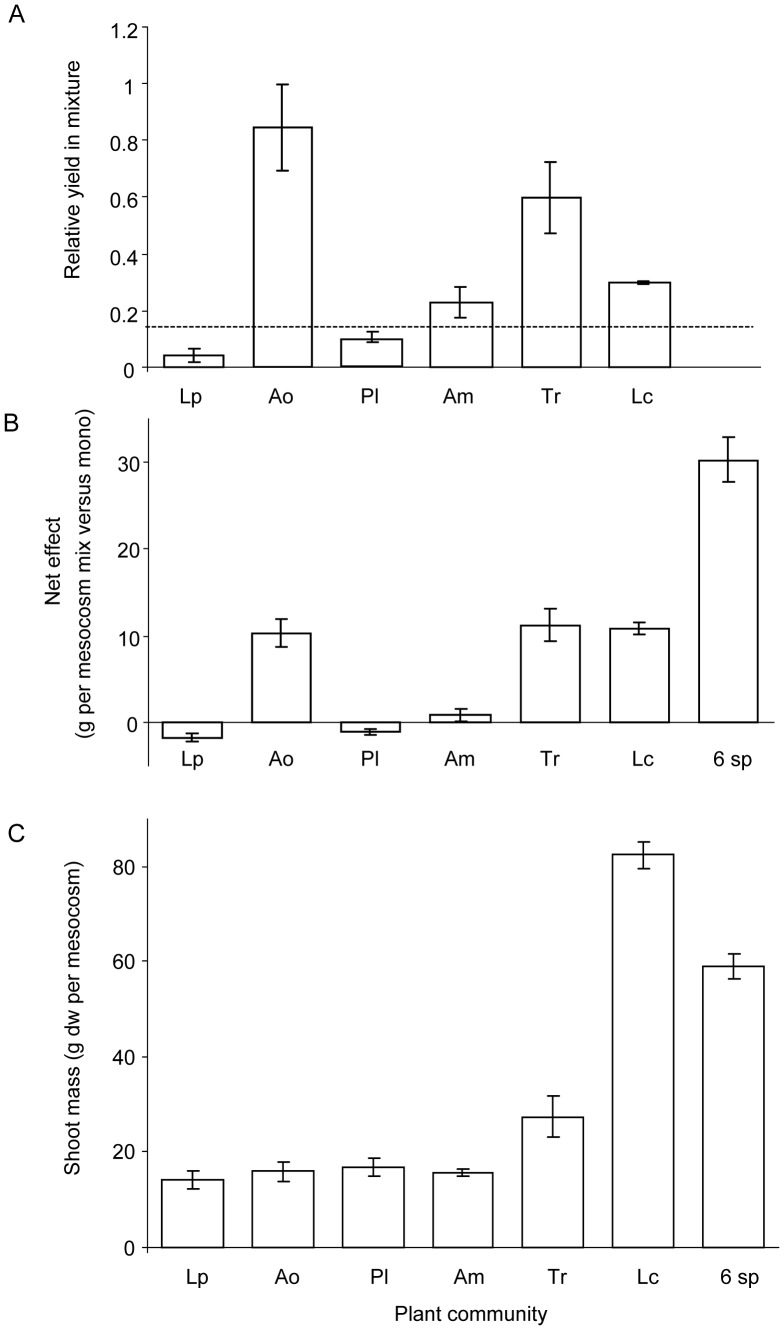
Plant species specific performance in species mixture and total biomass of monocultures and species mixtures. **Relative yield (A), net effect (B) and average shoot biomass (C) per mesocosm (38 × 38 cm) of each of the six species grown in monoculture and 6-species mixtures.** Bars are means ±1 SE (N = 4); the horizontal dotted line in panel A indicates 1/6^th^ of the yield. Species names are Tr = *Trifolium repens*, Lc = *Lotus corniculatus*, Pl = *Plantago lanceolata*, Ao = *Anthoxanthum odoratum*, Am = *Achillea millefolium*, Lp = *Lolium perenne*.

In mixed plant communities, all plant species, apart from *L. perenne*, performed better than in monoculture, as indicated by their positive relative yield (RY) (Fig. 1A); together, this resulted in a relative yield total (RYT) of 2.1±0.2 (Confidence Interval CI 1.5 to 2.7, N = 4). Individual plant species differed in their RY (K-W H = 20.9, P<0.001, N = 4), with *A. odoratum* and *T. repens* attaining the highest RY (Fig. 1A). However, plant species with a high RY did not necessarily contribute most to biomass production in mixtures, as RY reflects a biomass ratio and does not account for absolute biomass production. The absolute biomass contribution of the different plant species to overyielding of the 6-species mixtures was quantified as their net effect. We found that the net effect differed between species (F_5,15_ = 31.8, P<0.0001, N = 4), with *A. odoratum*, *T. repens* and *L. corniculatus* each contributing approximately one third (10 g per mesocosm) to the higher yield (Fig. 1B). Monoculture yield of *L. corniculatus* was higher than that of the 6-species mixtures, hence there was no transgressive overyielding. However, both *A. odoratum* and *T. repens* had much lower monoculture yields than the 6-species mixture, with their monoculture yield comparable to that of *P. lanceolata* and *A. millefolium* (Fig. 1C).

### ^13^C Enrichment in Vegetation and Soil

**Figure 2:**
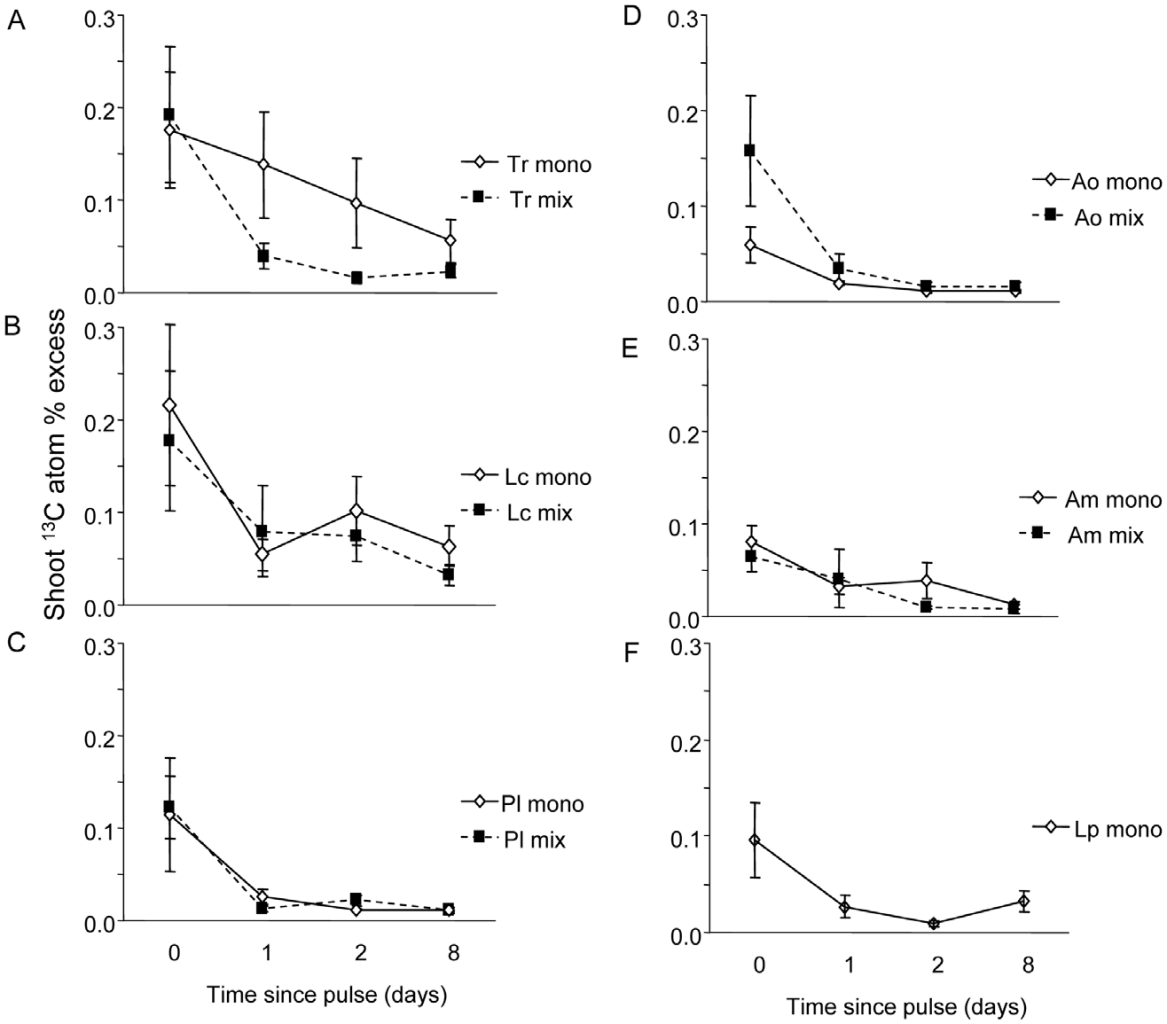
Enrichment of shoot tissue with ^13^C in individuals grown in monoculture (mono) or 6-species mixture (mix) at 2 h, 24 h, 48 h and 8 days after the ^13^C pulse. Species names are (A) Tr = *Trifolium repens*, (B) Lc = *Lotus corniculatus*, (C) Pl = *Plantago lanceolata*, (D) Ao = *Anthoxanthum odoratum*, (E) Am = *Achillea millefolium*, (F) Lp = *Lolium perenne*.

All species grown in monoculture and in the 6-species mixture were enriched in ^13^C at levels significantly above reference ‘background’ levels, as indicated by ^13^C atom % excess values (Fig. 2A–F; Fig. S1 A–F). The plant species with highest levels of enrichment were the legumes *T. repens* and *L. corniculatus* (Fig. 2A, B). For all species, ^13^C enrichment declined significantly over time (P<0.05), which was independent of species richness for *L. corniculatus*, *P. lanceolata*, *A. millefolium* and *A. odoratum*. In contrast, for *T. repens* there was a significant interaction with species richness (time × species richness interaction F_3,9_ = 4.69, P = 0.03, N = 4), with a faster decline in ^13^C enrichment occurring in the species mixture than in monoculture (Fig. 2A). For *A. odoratum*, ^13^C enrichment exhibited higher levels of enrichment in the 6-species mixture than in monoculture (F_1,3_ = 7.90, P = 0.067) (Fig. 2D). The %C in the vegetation was on average 41.2±0.2% (CI 40.8 to 41.5) across species and was not affected by whether species were grown in monoculture or 6-species mixture.

We found no statistically significant ^13^C enrichment in roots (average δ^13^C ranged from −25.2±0.7 ‰ (CI −26.6 to −23.9) to −24.1±0. 9 ‰ (CI −26.0 to −22.2) across sampling times), which was likely due to the dilution of the signal in mixed root samples with roots of varying age. We detected ^13^C enrichment of soil (Friedman Anova χ^2^ = 15.99, P<0.05, N = 4) and enrichment levels were higher immediately and one day after the pulse labelling than at two and eight days after the pulse. Soil ^13^C enrichment levels were low (average δ^13^C ranged from −25.8±0.1 ‰ (CI −26.0 to −25.5) at 24****h after the pulse to −26.13±0.08 ‰ (CI −26.3 to −26.0) at 48 hours after the pulse) and were unaffected by plant treatments.

### ^13^CO_2_ and Total CO_2_ Ecosystem Respiration

**Figure 3:**
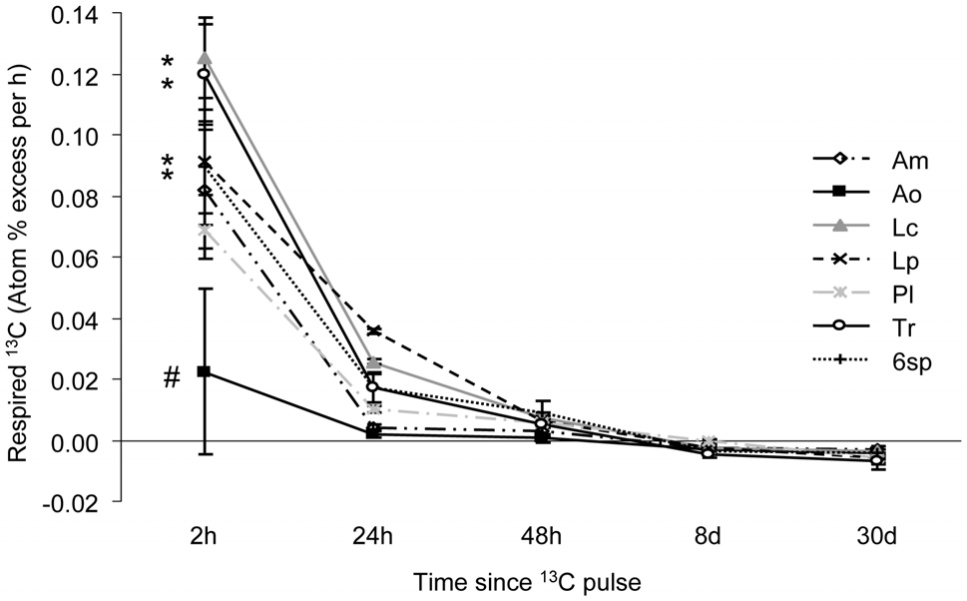
Enrichment of respired CO_2_ with ^13^C in monocultures and 6-species mixture (6sp) at 2 h, 24 h, 48 h, 8 and 30 days after the ^13^C pulse. Data points are means ±1 SE (N = 4). Communities with different symbols are significantly different at sampling time 2****h after the pulse. For species monoculture names see Fig. 1.

The rate of ^13^C loss through ecosystem respiration, in terms of enrichment of the atmosphere with ^13^C, declined strongly over time (F_4,72_ = 90.04, P<0.0001, N = 24), and showed an interaction with plant treatments (time × species richness interaction F_24,72_ = 2.03, P = 0.01, N = 4) (Fig. 3). This interactive effect was due to different rates of ^13^C respiration loss between the first two sampling points (2****h and 24****h after the pulse). Post-hoc tests revealed that monocultures of *L. corniculatus*, *T. repens* and *L. perenne* and 6-species mixtures had higher rates of ^13^C loss through respiration in terms of atmosphere enrichment with ^13^C than monocultures of *A. odoratum* at two hours after the pulse, while at subsequent sampling times the rate of ^13^C loss converged across all treatments. Total rates of ecosystem CO_2_ respiration differed significantly between the plant communities, being greatest in monocultures of *T. repens* compared to all other plant communities (Table 1). These differences were even more pronounced when expressed on a per unit aboveground dry weight biomass of the plant communities, with *T. repens* monocultures losing C through respiration at almost four times higher rates than 6-species mixtures.

**Table 1:** Rates of total ecosystem CO_2_ respiration per m^2^ and per aboveground biomass per m^2^ basis across the plant species monocultures and six species mixture.

Plant community	mean mg CO_2_-C/h/m^2^	−95% CI	+95% CI	mean mg CO_2_-C/h/m^2^/g dw	−95% CI	+95% CI
Tr	61.7	50.4	73.0	2.3	1.8	2.7
Lc	39.7	28.4	51.1	0.5	0.3	0.6
Lp	33.2	22.0	44.6	2.3	1.5	3.1
Am	25.5	14.1	36.8	1.6	0.9	2.3
Pl	24.3	13.1	35.7	1.5	0.8	2.1
Ao	20.6	9.2	32.0	1.3	0.6	2.0
6 sp	33.4	22.2	44.8	0.6	0.4	0.8

Means (in mg CO_2_-C/h/m^2^ and in mg CO_2_-C/h/m^2^/g aboveground dry weight) ±95% CI. Tr = *Trifolium repens*, Lc = *Lotus corniculatus*, Lp = *Lolium perenne*, Am = *Achilea millefolium*, Pl = *Plantago lanceolata*, Ao = *Anthoxanthum odoratum*, 6sp = mixture of the six species.

### Soil Nitrogen and pH

**Figure 4:**
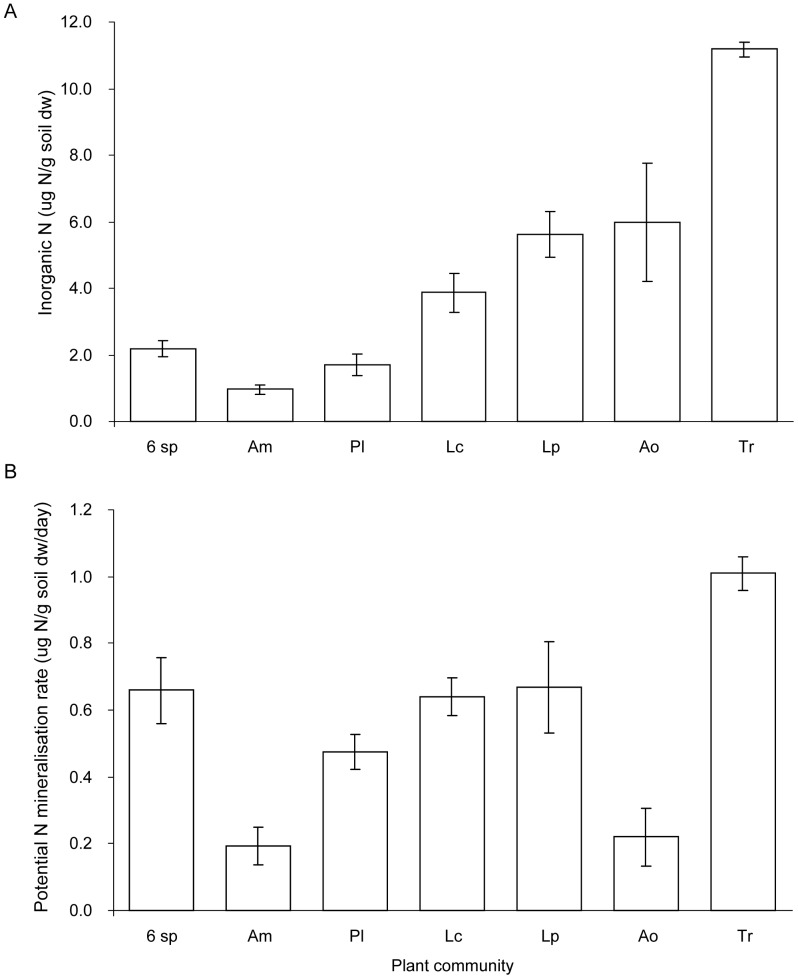
Soil mineral nitrogen status. **Total plant available inorganic N (A) and potential N mineralisation rate in µg per g soil dry weight per day (B) of soil from monocultures and 6-species mixtures (6 sp).** Bars are means ±1 SE (N = 4), for monoculture names see Fig. 1.

The availability of inorganic N at harvest was strongly affected by the plant treatments (F_6,18_ = 28.88, P<0.0001, N = 4; Fig. 4A). The highest concentration of inorganic N was found in soil of *T. repens* monocultures, intermediate values were found in soil of *A. odoratum*, *L. perenne* and *L. corniculatus* monocultures, and the lowest values in 6-species mixtures and monocultures of *P. lanceolata* and *A. millefolium* (Fig. 4A). Rates of potential N mineralisation were also strongly dependent on plant species treatments (F_6,18_ = 11.78, P<0.0001, N = 4; Fig. 4B). The highest rate of potential soil N mineralisation was found in monocultures of *T. repens*, the 6-species mixtures and monocultures of *L. corniculatus* and *L. perenne*, and lowest in soil taken from monocultures of *A. odoratum* and *A. millefolium* (Fig. 4B).

Soil pH differed significantly between plant species monocultures and 6-species mixtures (F_6,18_ = 18.28, P<0.0001, N = 4). Soils from the legumes *T. repens* and *L. corniculatus* had the lowest pH (5.66±0.06; *T. repens* CI 5.55 to 5.86 and for *L. corniculatus* CI 5.39 to 5.85), the grass *A. odoratum* the highest (6.57±0.14, CI 6.11 to 7.02), and 6-species mixtures had intermediate (5.88±0.03, CI 5.77 to 5.86) soil pH values.

### Vegetation C/N Ratio and NUE

**Figure 5:**
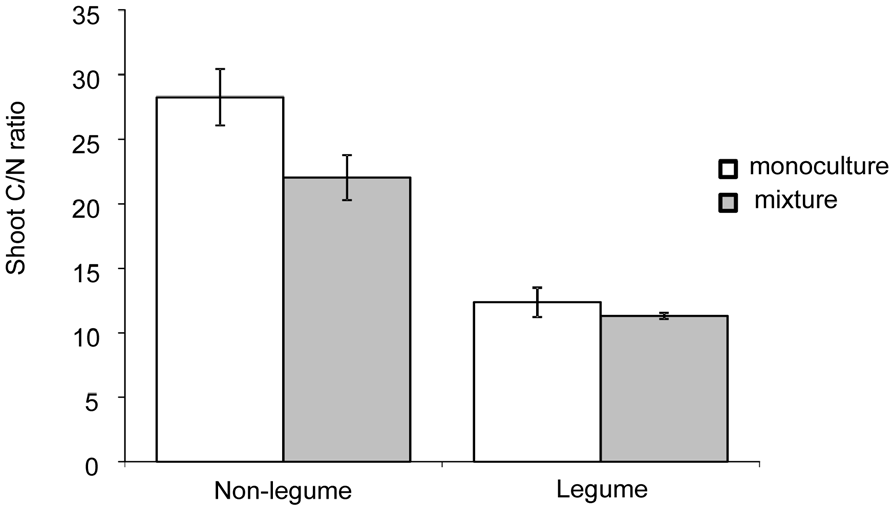
Average leaf C/N ration of non-legume and legume plant species grown in monoculture (white bars) and 6-species mixture (grey bars). Bars are means ±1 SE (N = 16 for Non-legume and N = 8 for Legume).

The average shoot C/N ratio (measured in the top 2 cm of plant shoots) of non-legume species was significantly lower when plants were grown in the 6-species mixture compared to when grown in monoculture (F_1,23_ = 6.6, P<0.05; in mixture CI 18.63 to 25.37 and in monoculture CI 24.75 to 31.75). In contrast, the C/N ratio of the legume species did not differ between monocultures or 6-species mixtures (F_1,11_ = 0.5, P>0.05; in mixture CI 10.14 to 12.44 and in monoculture CI 9.07 to 15.63), and was on average much lower than in the non-legume species (Fig. 5). We also found that the amount of aboveground biomass produced per unit of N in that plant material (i.e. their nutrient use efficiency NUE) differed significantly between assembled plant communities (Table 2). The NUE was lowest in monocultures of *T. repens*, intermediate in six species mixtures and monocultures of *L. corniculatus* and highest in monocultures of *A. odoratum*, *A. millefolium* and *P. lanceolata*.

**Table 2:** Nitrogen use efficiency at the community level (in aboveground biomass) across the plant species monocultures and six species mixture.

Plant community	NUE mean	−95% CI	+95% CI
Tr	25.0	23.4	26.7
Lc	49.7	41.8	57.7
Lp	85.8	37.1	134.6
Am	84.4	66.8	101.9
Pl	91.7	67.6	115.7
Ao	95.1	80.4	109.7
6 sp	42.6	38.7	46.6

NUE means (g dry weight/mg N) ±95% CI. Tr = *Trifolium repens*, Lc = *Lotus corniculatus*, Lp = *Lolium perenne*, Am = *Achilea millefolium*, Pl = *Plantago lanceolata*, Ao = *Anthoxanthum odoratum*, 6****sp = mixture of the six species.

## Discussion

In this study, we examined how intraspecific variation in short-term C translocation in grassland plant species is affected by being grown in mixed communities, and how this contributes to overyielding. We found that all plant species, with the exception of *L. perenne*, yielded more when grown in a six species mixture, but *T. repens* and *A. odoratum* benefitted most, with RY values of over 0.5. For these two plant species we also found that the ^13^C tracer enrichment in leaves and its translocation over time, was higher in plant individuals grown in 6-species mixture as opposed to when grown in monoculture. In particular, we found that the grass *A. odoratum* had higher levels of ^13^C enrichment following the application of the ^13^C pulse, while the legume *T. repens* showed a faster translocation of recently assimilated ^13^C; both these findings indicate faster C uptake and translocation in 6-species mixtures than in monocultures. Moreover, the release of recently assimilated ^13^C through respiration in terms of enrichment of the atmosphere with ^13^C from respiration was generally similar in monocultures and the 6-species mixture, while total standing biomass was higher in 6-species mixtures, suggesting higher C retention of assimilated C per unit biomass in species mixture. This idea is supported by the overall high rates of total CO_2_ respiration from *T. repens* monocultures which had low aboveground biomass and the low rates of total CO_2_ respiration loss from 6-species mixtures relative to their plant biomass.

The species with the greatest short-term ^13^C enrichment (*A. odoratum*) and ^13^C translocation from its leaves (*T. repens*) when grown in mixture relative to monoculture also benefited most from growing in species mixtures (high RY), and together contributed 2/3 of the absolute biomass increase in species mixtures (net effect). The other legume species used, *L. corniculatus*, also contributed significantly to the net effect on yield and had a high RY, indicating that it also benefited from growing in mixtures. However, we did not detect any effect of being grown in a mixture on leaf^ 13^C enrichment or ^13^C translocation rates in *L. corniculatus.* This indicates that other mechanisms are at play for this species, with a larger investment in stems (with low N content) rather than in leaves being one potential reason [16].

In grassland biodiversity experiments, the commonly observed increase of plant community biomass with species richness is usually strongly and positively related to the presence of legumes [11], [12], [21]. In these studies, the role of individual plants and species could not be addressed given their set-up with treatments using broad functional groups rather than individual species. In our experiment, legumes clearly played an important role in influencing non-legume species, with non-legume species having significantly lower shoot C/N ratios, i.e. containing relatively more N, when grown in 6-species mixtures than when grown in monoculture. We found larger plant biomass and higher leaf N concentrations in the 6-species mixtures; hence, our results support earlier findings that plants use a larger total soil N pool when in mixtures with legume species than when alone in monoculture [12], [15]. As a consequence, our results appear to be in contrast to other findings of higher N use efficiency, as indicated by lower C/N ratio of plant tissues and lower NUE at plant community level in species mixtures [12], [13]. The faster rate of C translocation in the grass species *A. odoratum* when grown in the species mixture with legumes may be explained by the higher N availability in soil and consequent improved total plant C assimilation and translocation activity.

Additional N input from N-fixers and resource use complementarity have been proposed as mechanisms underlying species richness-plant productivity relationships [1], [10], [11]. In our experiment, both legume species, *L. corniculatus* and *T. repens*, appeared to be very active in assimilating C, as indicated by high levels of ^13^C enrichment, which is known to promote atmospheric N fixation by legumes because N-fixing bacteria are fuelled by photosyntates [22]. Moreover, C translocation in *T. repens*, measured as change in ^13^C enrichment in its leaves over time, was greater in the mixture, suggesting enhanced C allocation to roots, although our ^13^C signal in roots was not strong enough to quantify this. It is unlikely that the faster ^13^C translocation was lost to respiration as the *T. repens* monocultures respired CO_2_ at much faster rate than plant species mixtures. Given that aboveground biomass in 6-species mixture was basically composed of 1/3^rd^ of each *T. repens*, *A. odoratum* and *L. corniculatus*, as determined at final harvest, one could expect respiratory loss on a per unit aboveground biomass basis in the species mixture being the sum of 1/3^rd^ of the C respiration loss rates per unit aboveground biomass of *T. repens*, *A. odoratum* and *L. corniculatus*. However, in species mixture the measured C respiration loss rate per unit aboveground biomass was less than half the expected loss rate, suggesting enhanced carbon use efficiency in plant species mixtures. In addition to larger N input, the mesocosms with 6-species mixture lost less N through leaching compared to monocultures [23]. Previous studies have reported complementarity between legumes and C4 grasses [12], and between legumes and tall herbs [24], whereas our results suggest complementarity between legumes and C3 grasses. The explanation of the observation that in the different studies legumes were complementary to different plant groups (i.e. to C4 grasses, or to tall herbs, or to C3 grasses) may be different overruling underlying mechanisms. For example in mixtures where legumes and C4 grasses are combined complementarity in peak growing season may be the overruling factor in overyielding, which may be less at play when legumes and C3 grasses are combined [4], [12].

The reason why the legume species had faster C translocation and benefited from growing in mixtures is not clear, but may be related to soil pH, which was lowest in the legume monocultures and may have suppressed N fixation rates [25]. Also the lower soil mineral N availability in species mixtures as compared to in monocultures of *T. repens* may have promoted N fixation rates, and with it associated high rates of C allocation to roots in *T. repens* grown in plant species mixtures [26]. The faster rate of translocation of C in *T. repens* in species mixtures indeed suggests a potential change in C sink strength of plant roots in species mixtures. Apart from root inhabiting rhizobia also arbuscular mycorrhizal fungi (AMF) may have facilitated faster ^13^C translocation in *T. repens* leaves to belowground in species mixtures as mycorrhiza can stimulate the C sink strength and rates of N fixation in legume roots [22]. In our plant communities, we found that plant species richness and *A. odoratum* presence related positively to the abundance of AMF in soil [27]. If *A. odoratum* stimulates C sink strength in roots of *T. repens* through a common AMF network, and if AMF promote N uptake in *A. odoratum*, this might explain why *A. odoratum* and *T. repens* performed especially well in species mixtures i.e. by indirect, soil biota mediated, reciprocal benefit. In an earlier experiment, using the same *in situ* labelling approach, we found that AMF are a strong C sink and show significant ^13^C enrichment 24 h after labelling, which indicates that they act at timescales that correspond with the rate of decrease of the ^13^C tracer in plant shoots [28].

The higher leaf C/N ratios measured in monocultures compared with the 6-species mixture, indicate that N limitation of non-legume species in monocultures was an important factor in their lower yielding. The overall low yield of *L. perenne*, *P. lanceolata* and *A. millefolium* in monoculture and mixture may be due to the combination of factors, such as low soil nutrient availability, soil pathogens and competition, so that C loss to pathogens and herbivores was not compensated for [29], [30].

By tracing C from the atmosphere into individual plant species within plant species grown in monoculture and 6-species mixture, we found that short-term plant C translocation is accelerated in plant individuals of legume and C3 grass species when plants are grown in mixtures, potentially through interaction with soil biota. Moreover this short-term response was strongly positive related to overyielding in species mixtures measured at longer-term. These results show a mechanistic coupling between intraspecific plant carbon cycling and community level productivity.

## Materials and Methods

### Experimental Design

Plant communities were established in outdoor mesocosms in August 2006. The mesocosms (38 × 38 cm, 30 cm deep) comprised high-density polypropylene pots with a 10-cm bottom layer of limestone chippings, filled with 20 cm of soil and placed on a polypropylene saucer [23]. Soil was taken from a permanent grassland with a history of fertiliser application, at the University of Newcastle-upon-Tyne Farm, Nafferton, Northumberland, UK (54°1′ N, 0°23′ W). The soil was collected from the top 20 cm layer after stripping off the vegetation. Prior to filling mesocosms, all stones and visible roots were removed by hand and the soil was mixed [23]. The soil was a free-draining alluvial sandy loam soil (3% clay, 42% loam, 55% sand), as determined using a particle size analyzer (Mastersizer 2000, Malvern Instruments Ltd, Malvern, UK). At the start of the experiment, total available inorganic N (KCl extract) was 16.5±0.4 mg kg^−1^ soil dry weight, organic matter content 4.3±0.1% as determined by loss on ignition at 550°C, and soil was pH 5.8±0.1 [21].

The experiment was set up at the Lancaster University Hazelrigg Field Station (54°1′ N, 2°46′ W; mean annual temperature 9°C and mean annual precipitation 1050 mm) in a complete random block design. The experiment comprised four blocks, each containing one monoculture of each of the six plant species and a mixture of the six species all grown together, totalling 7 communities × 4 reps = 28 mesocosms. Plants were planted in mesocosms at a constant total plant density of 36 individuals per mesocosm, with 6 individuals per species planted in the mixtures so that individuals of each species were neighboured by individuals of other species. Plants were planted in six rows with in each row six individuals, one of each plant species in the case of plant species mixtures. Our species pool consisted of common British grassland plants: two grasses *Lolium perenne* L. (Lp) and *Anthoxanthum odoratum* L. (Ao), two forbs *Plantago lanceolata* L. (Pl) and *Achillea millefolium* L. (Am), and two legume species *Trifolium repens* L. (Tr) and *Lotus corniculatus* L. (Lc) [31], [32]. All plants were grown from seed by germinating surface-sterilized seeds (using diluted sodium hypochlorite) in Petri dishes with filter paper soaked in de-mineralised water at room temperature. Germinated seeds were transferred to plug trays with autoclaved sterilized soil and grown in a glasshouse for eight weeks. Seedlings were subsequently acclimatised outside for one week and then planted in the mesocosms. After one year in August 2007, above-ground vegetation was clipped to 2 cm above the soil surface, and plant communities were left to re-grow. In order to maintain the original species composition, mesocosms were weeded of unwanted species. Mesocosms received no fertiliser throughout the experiment and were watered during summer months as required.

### ^13^CO_2_ Pulse Labelling

To investigate the fate of recently plant assimilated C, a ^13^CO_2_ pulse-chase assay was performed according to the method of Ostle et al. [20], [33], and as used by De Deyn et al. [28]. The vegetation was subjected to a ^13^C-CO_2_ pulse-chase treatment in July 2008, followed by sampling of individual plant species. In brief, in each mesocosm vegetation was exposed to an air stream to which ^13^C labelled CO_2_ (99 atom % ^13^C enriched) was added and passed through a 19 l transparent acrylic chamber (30 cm diameter, 35 cm height) at flow rates of 3 l per minute at a concentration of 500 ppm CO_2_. The pulse labelling system simultaneously provided 14 chambers with ^13^C enriched CO_2_. Therefore we pulsed 14 of the 28 mesocosms across the four blocks (i.e. half of all the mesocosms in each block) during 30 minutes, then moved the pulse chambers to the other half of the mesocosms in each block for 30 minutes. We repeated this cycle five times so that each mesocosm was exposed to an equal ^13^CO_2_ pulse for 2.5 hours between 11 am and 4 pm on 16th July 2008. Average air temperature during pulsing was 15°C, soil temperature 16°C and PAR was 670±130 µmol m^−2^ s^−1^.

### Plant, Soil and Respired CO_2_ Sampling

Plant and soil matter was sampled immediately before ^13^CO_2_ labelling and at 2, 24 and 48 hours, and 8 days after labelling. At each sampling, shoot material from each of the six plant species was sampled across all mesocosms. Vegetation was sampled by snipping 2 cm long leaf tips from undamaged plants or a leaflet of 3 sub-leaves for *T. repens*. Samples of each plant species from each mesocosm were put in individual eppendorf tubes and were immediately frozen at −20°C. Soil samples were collected for root, inorganic and mineralisable N measurements from the rhizosphere by taking a single core (3.4 cm diameter, 10 cm deep). Soil cores and vegetation samples were taken from a different quarter of the labelled area within each mesocosm at each sampling. All plant species sampled were present in all quarters of the labelled area, with the labelled area being the central area of diameter 30 cm (i.e. chamber diameter) within the square pot area. Release of recent assimilated ^13^C through ecosystem CO_2_ respiration (soil, root and shoot) was assessed by covering the vegetation with dark chambers and head space samples were collected immediately after covering and after 30 minutes and 1 hour through a septum (Suba Seal) fitted in the chamber wall prior to covering the vegetation with the chamber. Samples were stored in 12 ml exetainers (labco Ltd UK) at sampling temperature.

Total above-ground vegetation was harvested from each mesocosm at the end of August 2008 by clipping all shoot material above the soil surface. Vegetation was dried at 70°C for 48 h and weighed per species and per mesocosm. Relative yield (RY) of each species was calculated within each block as the species biomass in mixture divided by the species biomass in monoculture (for species i: RY_i_ = Y_i_/M_i_), and relative yield total (RYT) by summing the RY of all species in the mixed plant community whereby RYT >1 was qualified as overyielding [10], [34]. The net effect or net yield (net Y) of each species quantifies the biomass contribution of each plant species to the extra biomass in the species mixture as compared to the average monoculture biomass. We calculated the net Y within each block as the observed yield (Y_O_) in the species mixture minus the expected yield (Y_E_), with the expected yield being the monoculture yield of the species divided by the number of species in the mixture (net Y_i_ = Y_Oi_−Y_Ei_ = Y_Oi_−(M_i_/6)) [35].

### Stable Isotope Analyses and Plant C and N Concentration

Enrichment of ^13^C in plant tissues (leaf and root) and soil was determined using freeze-dried (Gilchrist, Germany) and finely ground sample material (<50 µm). Sample was weighed into tin cups, and analysed for total C, ^13^C/^12^C isotopic ratio, % C and % N using a Flash EA 1112 Series elemental analyser (Thermo Electron Corporation, Bremen, Germany) coupled with a Deltaplus Advantage isotope ratio mass spectrometer (IRMS, Thermo Finnigan, Bremen, Germany). Enrichment of ^13^C is expressed as ^13^C atom % excess according to Boutton [36] with atom % excess = atom % enriched sample – atom % background sample (i.e. before labelling), in which atom % = [R_sample_/(R_sample_ +1)] × 100 and R_sample_ = ^13^C/^12^C ratio measured by IRMS. ^13^C/^12^C isotopic ratios of respiration samples were also determined using a Gas-bench II connected to a Deltaplus Advantage isotope ratio mass spectrometer (both Thermo Finnigan, Bremen, Germany) and CO_2_ concentrations determined by Gas Chromatography (Agilent, Autosystem xl).

### Soil Nitrogen, Potential Nitrogen Mineralisation Rate and pH

Total plant available inorganic N concentrations (NH_4_^+^ + NO_3_^−^) and rates of potential N mineralisation were determined on soil samples collected immediately after harvesting all the vegetation. Total available inorganic N was determined in KCl extracts of subsamples of 10 g of fresh sieved soil using standard colorimetric autoanalyser procedures [37] on a continuous flow autoanalyser (Bran and Luebbe, Northampton, UK). To evaluate potential rates of N mineralisation, inorganic N (NH_4_^+^ + NO_3_^−^) was also determined after incubation of 10 g subsamples of fresh soil at 20°C for 14 days and the potential mineralisation rate was calculated as inorganic N concentration after incubation, minus that at the start, divided by the number of days of incubation. Soil pH was determined on water extracts using a 12.5 gram fresh sieved soil subsample in 50 ml of demineralised water and a pH meter.

### Data Analysis

We tested for the effects of plant species identity on biomass yield in the 6-species mixtures using Generalised Linear Mixed Model (GL Mixed Model), with block as a random factor and species identity as fixed factor. We used Repeated Measures Analysis of Variance (ANOVA) to test the effect of plant species richness on ^13^C enrichment, on CO_2_ respiration rate and on %C in vegetation for each species, with block as a random factor and species richness (1 or 6) as fixed, and sampling time as a repeat factor. In a similar way, we tested the effect of plant composition (monoculture identity and 6-species mixture) on ^13^C respiration loss using Repeated Measures ANOVA with plant treatment as a fixed factor, block as a random factor, and sampling time as a repeat factor. Plant treatment effects on soil inorganic N, potential N mineralisation rate, shoot biomass, shoot C/N ratio of non-legume and legume species and soil pH were tested using a GL Mixed Model with block as random and plant community composition (the six monocultures and 6-species mixtures) as fixed factor. Differences between treatment levels were tested using Tukey HSD post-hoc test. Enrichment with ^13^C and shoot C/N in *L. perenne* in mixtures could not be determined due to the very low abundance of this species in mixtures. Species identity effects on relative yield and plant community composition on ^13^C enrichment in soil were tested using non-parametric Kruskal-Wallis and Mann-Whitney U tests, and time effects on soil ^13^C enrichment were tested using Friedmann ANOVA for multiple dependent samples. Prior to statistical analysis, data of ^13^C enrichment in plants and air and shoot C/N ratios were log transformed, and total inorganic N data were square root transformed. We used the software STATISTICA for our statistical analysis.

## Supporting Information

Figure S1 **Enrichment of shoot tissue with ^13^C in individuals grown in monoculture (mono) or 6-species mixture (mix) at 2 h, 24 h, 48 h and 8 days after the ^13^C pulse with test statistics per plant species.** Species names are (A) Tr = *Trifolium repens*, (B) Lc = *Lotus corniculatus*, (C) Pl = *Plantago lanceolata*, (D) Ao = *Anthoxanthum odoratum*, (E) Am = *Achillea millefolium*, (F) Lp = *Lolium perenne*.
(TIF)

## References

[1] Hooper DU, Chapin FS, Ewel JJ, Hector A, Inchausti P, et al (2005) Effects of biodiversity on ecosystem functioning: A consensus of current knowledge. Ecol Monographs 75: 3–35.

[2] Cardinale BJ, Wright JP, Cadotte MW, Carroll IT, Hector A, et al (2007) Impacts of plant diversity on biomass production increase through time because of species complementarity. Proc Natl Acad Sci U S A 104: 18123–18128. 17991772 10.1073/pnas.0709069104 PMC2084307

[3] Berendse F (1983) Interspecific competition and niche differentiation between *Plantago lanceolata* and *Anthoxanthum odoratum* in a natural hayfield. J Ecol 71: 379–390.

[4] Hooper DU, Vitousek PM (1998) Effects of plant composition and diversity on nutrient cycling. Ecol Monographs 68: 121–149.

[5] Spehn EM, Joshi J, Schmid B, Diemer M, Korner C (2000) Above-ground resource use increases with plant species richness in experimental grassland ecosystems. Funct Ecol 14: 326–337.

[6] Weigelt A, Bol R, Bardgett RD (2005) Preferential uptake of soil nitrogen forms by grassland plant species. Oecologia 142: 627–635. 15549402 10.1007/s00442-004-1765-2

[7] Harrison KA, Bol R, Bardgett RD (2007) Preferences for different nitrogen forms by coexisting plant species and soil microbes. Ecology 88: 989–999. 17536714 10.1890/06-1018

[8] von Felten S, Hector A, Buhmann N, Niklaus PA, Schmid B, et al (2009) Belowground nitrogen partitioning in experimental plant communities of varying species richness. Ecology 90: 1389–1399. 19537558 10.1890/08-0802.1

[9] Schnitzer SA, Klironomos JN, HilleRisLambers J, Kinkel LL, Reich PB, et al (2011) Soil microbes drive the classic plant diversity-productivity pattern. Ecology 92: 296–303. 21618909 10.1890/10-0773.1

[10] Fridley JD (2001) The influence of species diversity on ecosystem productivity: how, where, why and when? Oikos 93: 514–526.

[11] HilleRisLambers J, Harpole WS, Tilman D, Knops J, Reich PB (2004) Mechanisms responsible for the positive diversity-productivity relationship in Minnesota grasslands. Ecol Letters 7: 661–668.

[12] Fargione J, Tilman D, Dybzinski R, HilleRisLambers J, Clark C, et al (2007) From selection to complementarity: shifts in the causes of biodiversity-productivity relationships in a long-term biodiversity experiment. Proc Roy Soc B Biol Sci 274: 871–876. 10.1098/rspb.2006.0351 PMC2093979 17251113

[13] van Ruijven J, Berendse F (2005) Diversity-productivity relationships: Initial effects, long-term patterns, and underlying mechanisms. Proc Natl Acad Sci U S A 102: 695–700. 15640357 10.1073/pnas.0407524102 PMC545547

[14] Marquard E, Weigelt A, Roscher C, Gubsch M, Lipowsky A, et al (2009a) Positive biodiversity-productivity relationship due to increased plant density. J Ecol 97: 696–704.

[15] Gubsch M, Buchmann N, Schmid B, Schulze ED, Lipowsky A, et al (2011) Differential effects of plant diversity on functional trait variation of grass species. Annals Botany 107: 157–169. 10.1093/aob/mcq220 PMC3002477 21068024

[16] Roscher C, Schmid B, Buchmann N, Weigelt A, Schulze ED (2011) Legume species differ in the responses of their functional traits to plant diversity. Oecologia 165: 437–452. 20680645 10.1007/s00442-010-1735-9

[17] Ward SE, Bardgett RD, McNamara NP, Ostle NJ (2009) Plant functional group identity influences short-term peatland ecosystem carbon flux: evidence from a plant removal experiment. Funct Ecol 23: 454–462.

[18] Harris D, Pacovsky RS, Paul EA (1985) Carbon economy of soybean-Rhizobium-Glomus associations. New Phytologist 101: 427–440. 10.1111/j.1469-8137.1985.tb02849.x 33874245

[19] Fridley JD, Grime JP (2010) Community and ecosystem effects of intraspecific genetic diversity in grassland microcosms of varying species diversity. Ecology 91: 2272–2283. 20836449 10.1890/09-1240.1

[20] Ostle N, Whiteley AS, Bailey MJ, Sleep D, Ineson P, et al (2003) Active microbial RNA turnover in a grassland soil estimated using a ^13^CO_2_ spike. Soil Biol Biochem 35: 877–885.

[21] Tilman D, Reich PB, Knops J, Wedin D, Mielke T, et al (2001) Diversity and productivity in a long-term grassland experiment. Science 294: 843–845. 11679667 10.1126/science.1060391

[22] Kaschuk G, Kuyper TW, Leffelaar PA, Hungria M, Giller KE (2009) Are the rates of photosynthesis stimulated by the carbon sink strength of rhizobial and arbuscular mycorrhizal symbioses? Soil Biol Biochem 41: 1233–1244.

[23] De Deyn GB, Quirk H, Yi Z, Oakley S, Ostle NJ, et al (2009) Vegetation composition promotes carbon and nitrogen storage in model grassland communities of contrasting soil fertility. J Ecol 97: 864–875.

[24] Marquard E, Weigelt A, Temperton VM, Roscher C, Schumacher J, et al (2009b) Plant species richness and functional composition drive overyielding in a six-year grassland experiment. Ecology 90: 3290–3302. 20120799 10.1890/09-0069.1

[25] Zahran HH (1999) Rhizobium-legume symbiosis and nitrogen fixation under severe conditions and in an arid climate. Microbiol Molecular Biol Rev 63: 968–989. 10.1128/mmbr.63.4.968-989.1999 PMC98982 10585971

[26] Hartwig UA (1998) The regulation of symbiotic N2 fixation: a conceptual model of N feedback from the ecosystem to the gene expression level. Perspectives Plant Ecol Evol Syst 1: 92–120.

[27] De Deyn GB, Quirk H, Bardgett RD (2011) Plant species richness, identity and productivity differentially influence key groups of microbes in grassland soils of contrasting fertility. Biol Letters 7: 75–78. 10.1098/rsbl.2010.0575 PMC3030891 20685699

[28] De Deyn GB, Quirk H, Oakley S, Ostle NJ, Bardgett RD (2011) Rapid transfer of photosynthetic carbon through the plant-soil system in differently managed species-rich grasslands. Biogeosci 8: 1131–1139.

[29] Petermann JS, Fergus AJF, Turnbull LA, Schmid B (2008) Janzen-Connell effects are widespread and strong enough to maintain diversity in grasslands. Ecology 89: 2399–2406. 18831160 10.1890/07-2056.1

[30] De Deyn GB, Raaijmakers CE, van der Putten WH (2004) Plant community development is affected by nutrients and soil biota. J Ecol 92: 824–834.

[31] Grime JP, Hunt R (1975) Relative growth-rate – its range and adaptive significance in a local flora. J Ecol 63: 393–422.

[32] Rodwell JS (1992) British plant communities: grasslands and montane communities. Cambridge University Press, Cambridge, UK.

[33] Ostle N, Briones MJI, Ineson P, Cole L, Staddon P, et al (2007) Isotopic detection of recent photosynthate carbon flow into grassland rhizosphere fauna. Soil Biol Biochem 39: 768–777.

[34] de Wit CT, van den Bergh JP (1965) Competition between herbage plants. Netherlands J Agric Sci 13: 212–221.

[35] Loreau M, Hector A (2001) Partitioning selection and complementarity in biodiversity experiments. Nature 412: 72–76. 11452308 10.1038/35083573

[36] Boutton TW (1991) Stable carbon isotope ratios of natural materials:1. Sample preparation and mass spectrometric analysis. In: Coleman DC editor. Carbon Isotope Techniques. Academic press Ltd, London. 155–186.

[37] Ross DJ (1992) Influence of sieve mesh size on estimates of microbial carbon and nitrogen by fumigation-extraction procedures in soils under pasture. Soil Biol Biochem 24: 343–350.

