# Supplementary figures and images for: Increased Plant Carbon Translocation Linked to Overyielding in Grassland Species Mixtures
Source: PLoS One. 2012 Sep 25;7(9):e45926. doi: 10.1371/journal.pone.0045926 (PMC3457971; doi:10.1371/journal.pone.0045926)

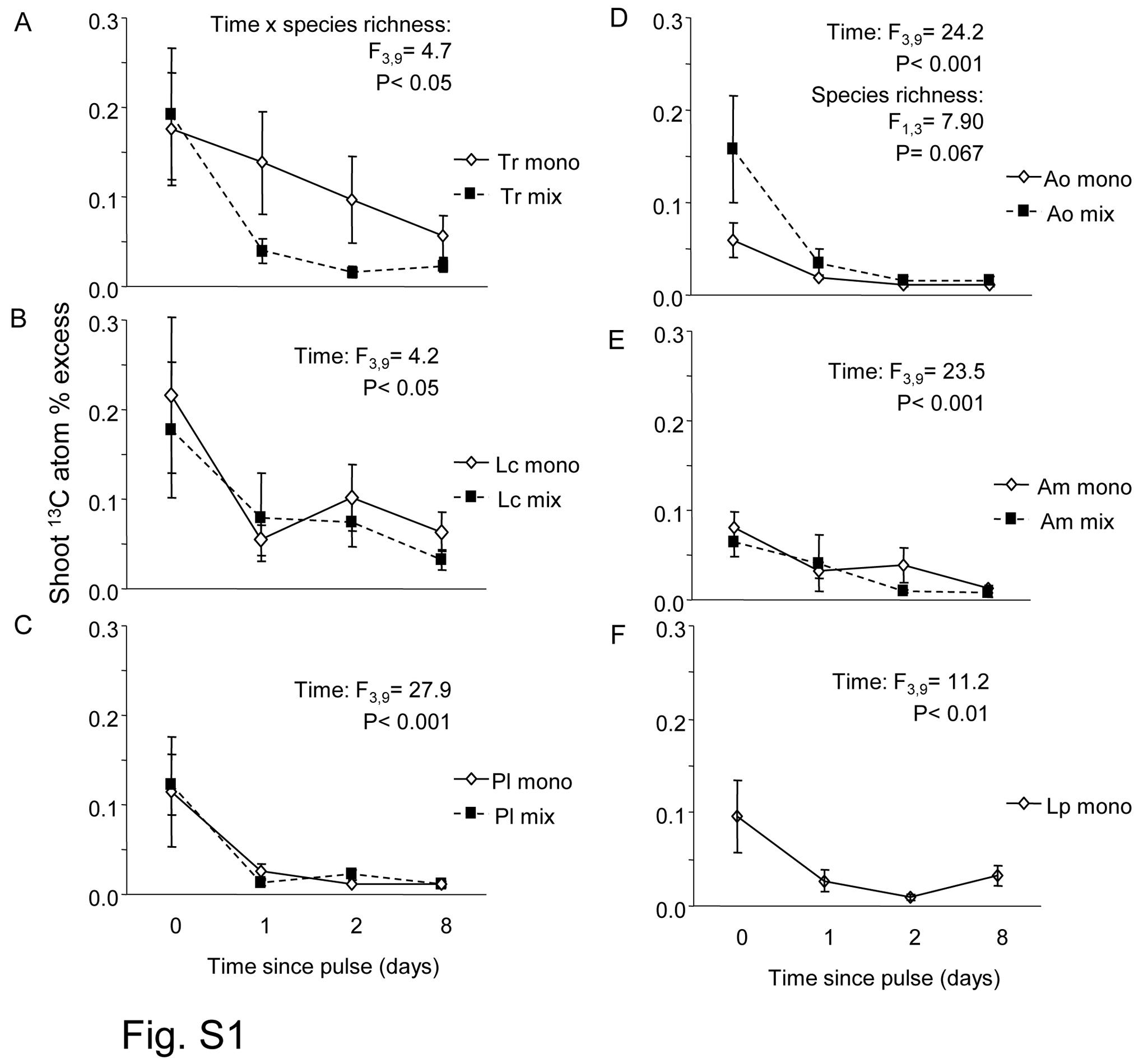

Supplement: Figure S1 — Enrichment of shoot tissue with 13C in individuals grown in monoculture (mono) or 6-species mixture (mix) at 2 h, 24 h, 48 h and 8 days after the 13C pulse with test statistics per plant species. Species names are (A) Tr = Trifolium repens, (B) Lc = Lotus corniculatus, (C) Pl = Plantago lanceolata, (D) Ao = Anthoxanthum odoratum, (E) Am = Achillea millefolium, (F) Lp = Lolium perenne. (TIF) [file pone.0045926.s001.tif]
